# Knowledge and Attitude towards Retrograde Peri-Implantitis among Italian Implantologists: A Cross-Sectional Survey

**DOI:** 10.3390/ijerph17228356

**Published:** 2020-11-12

**Authors:** Bianca Di Murro, Nicola Pranno, Andrea Raco, Roberto Pistilli, Giorgio Pompa, Piero Papi

**Affiliations:** 1 Department of Oral and Maxillo-Facial Sciences, “Sapienza” University of Rome, 00161 Rome, Italy; bianca.dimurro@uniroma1.it (B.D.M.); nicola.pranno@uniroma1.it (N.P.); a.raco@hotmail.it (A.R.); giorgio.pompa@uniroma1.it (G.P.); 2 Oral and Maxillofacial Unit, San Camillo Hospital, 00152 Rome, Italy; r_pistilli@libero.it

**Keywords:** retrograde peri-implantitis, peri-implantitis, apical peri-implantitis, survey, implant periapical lesion

## Abstract

Background: Retrograde peri-implantitis (RPI) is a pathological entity with an unclear etiology (e.g., overheating during implant insertion, residual infection of the tooth replaced by the implant or the endodontic lesion of neighboring teeth) and an extremely low prevalence and has been scarcely investigated. Therefore, the aim of this cross-sectional survey was to evaluate the knowledge and attitude of Italian implantologists regarding RPI. Methods: An anonymous questionnaire was sent via email to implantologists randomly selected, including a section about demographic information and questions related to RPI origin, radiographic representation, symptoms and treatment options. All questions were multiple answer and close-ended. Binomial logistic regression was performed to investigate the relationship between correct answers and the following independent variables: age, years of experience and number of dental implants placed per year. Results: In total, 475 implantologists completed the questionnaire, with a response rate of 46.3%. Based on the results of the study, incorrect answers were associated with less experienced participants (<80 implants/year) for all questions evaluated, with the exception of treatment strategies. Furthermore, 26.7% of the survey takers did not recognize radiographic representation of RPI and 35.5% picked “implant removal” when asked about treatment modality. Conclusions: The majority of participants were able to recognize symptoms and indicated the probable causes of RPI; however, around 30% of them showed very limited knowledge of available management strategies.

## 1. Introduction

During the last decades, the replacement of missing teeth with dental implants has become a successful and predictable option for clinicians [1,2,3,4]. However, peri-implantitis, characterized by bleeding and/or suppuration on gentle probing and progressive loss of supportive bone around dental implants, represents a well-known cause of implant failure [5,6,7,8]. There is another pathological entity, described as retrograde peri-implantitis (RPI) [9,10,11], which represents a less-known disease [12]. This lesion appears radiographically as a radiolucent area around the apical part of the implant. While in peri-implantitis, lesion bone resorption follows a corono-apical trend, on the contrary, RPI presents an apico-coronal pattern, with or without clinical signs of inflammation [13]. The diagnosis of RPI is based on clinical and radiological findings and commonly occurs within the first 8 weeks after implant placement [14,15,16].

This implant disease was first described by McAllister et al. [17] in 1992 and seems to have an incidence ranging from 0.26% to 1.86% [18,19,20,21], significantly lower than marginal peri-implantitis. Although the etiology of this lesion is still unclear [22], according to several authors, the causes more accredited could be the residual infection/inflammation of the tooth replaced by the implant or the endodontic lesion of neighboring teeth [13,23,24]. Among other factors hypothesized, there are bone overheating or implant insertion in a site with residual granulomatous/cystic lesions [22].

Over the years, the administration of simple questionnaires has proven to be an effective and economic way to investigate the perception and habits of clinicians on specific matters [25,26,27]. Since there were just a few case reports published on RPI by Italian researchers [28,29,30,31], investigating its knowledge in an Italian sample of implantologists could be interesting. Furthermore, since this condition is extremely rare [18,19,20,21] and the majority of current available literature has been produced in specialized university settings by experienced research groups, it would be interesting to further explore the effect of age, years of experience and number of dental implants placed on the topic.

Therefore, the aim of this cross-sectional survey was to evaluate the knowledge and attitude of Italian implantologists regarding retrograde peri-implantitis. The secondary aim was to explore the association between age, years of experience in dentistry and number of dental implants placed per year on the implantologists’ knowledge of retrograde peri-implantitis.

## 2. Materials and Methods

### 2.1. Study Design

To address the research purpose, the authors developed and implemented a cross-sectional online survey, conducted at the Department of Oral and Maxillo-Facial Sciences, at “Sapienza” University of Rome from January to March 2020. The study was reported in accordance with the STROBE statement. All questionnaires were anonymous, and no personal data of participants were collected; therefore, ethical approval was not required based on the recommendations of our Institution Review Board (“Sapienza” University of Rome).

### 2.2. Study Population

An anonymous questionnaire was sent via email to implantologists randomly selected from the official register of oral surgery and implantology Italian scientific societies. Only dentists placing dental implants on a regular basis were enrolled in this survey. To limit the survey to implantologists, the first question was: “Do you perform implant dentistry on a regular basis?”; in case of negative answer, the respondent was excluded from the study. The questionnaire was sent to a randomly selected sample of 1026 implantologists, extrapolated using a randomization program from the complete email list of the scientific societies’ members.

### 2.3. Survey

The invitation emails contained an individual link to a web-based questionnaire, which could only be answered once. Reminder emails were sent once/week for the entire length of the study period in order to increase participation. No monetary incentive was given to participants. Since the questionnaire was anonymous, no personal data of participants were collected. The questionnaire included a section about demographic information, while the main body of the survey included questions related to RPI origin, radiographic representation, symptoms and treatment options.

In addition, the first question of the survey focused on the source of information: “Where did you learn about RPI?”, while the last was “Have you have ever treated an RPI case?”.

All questions were multiple answer and close-ended but had a different evaluation system; in three cases (questions #2, #4 and #5), more than one answer was allowed (Supplementary 1).

### 2.4. Statistical Analysis

Data were evaluated using standard statistical analysis software (version 20.0, Statistical Package for the Social Sciences, IBM Corporation, Armonk, NY, USA). A database was created using Excel (Microsoft, Redmond, WA, USA). Descriptive statistics were presented as numbers and percentages for each variable.

All variables included were categorical and were compared using the chi-squared or Fisher’s test, as appropriate. Four dichotomous dependent variables were analyzed, evaluating correct answers for etiology, symptoms, radiographic representation and treatment strategies of retrograde peri-implantitis.

Multivariable analysis adjusted for sex was performed to investigate the relationship between the dependent variables and the following independent variables: age (≤30; 31–44; ≥45), years of experience in dentistry (<5–9; 10–19; >19) and number of dental implants placed per year (<20; 20–80; >80). Results of multivariable analysis were presented as Odds Ratios (OR) with 95% confidence intervals. In each test, the cut-off for statistical significance was *p* ≤ 0.05.

## 3. Results

Out of the 1026 emails sent, 475 implantologists completed the questionnaire, with a response rate of 46.3%. The sample was composed, by a vast majority, of men (88.9%), with more than 45 years of age (55.8%), while 44.7% of the survey takers had >19 years of experience in implant dentistry and 49.1% placed from 20 to 80 implants per year.

**Figure 1:**
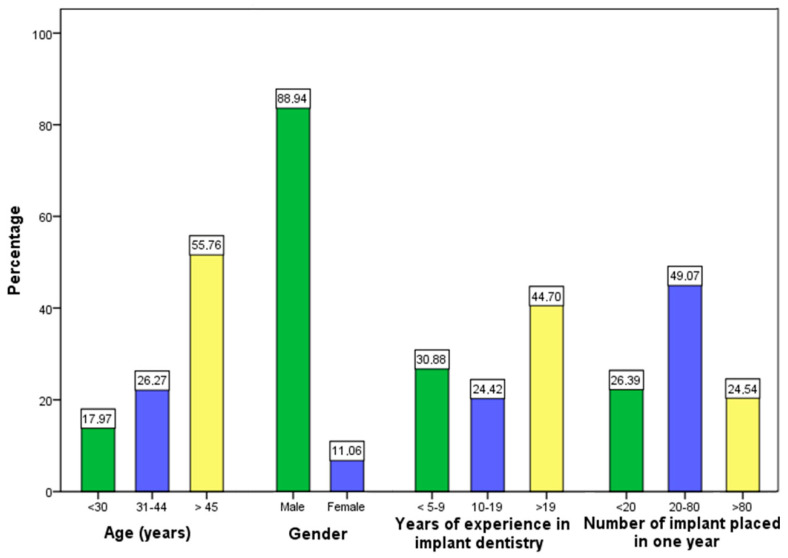
The distribution of the sample according to age, gender, years of experience in implant dentistry and number of implant placed in one year.

The accurate distribution of sample according to age, gender, years of working experience in implant dentistr, and number of implants placed per year is summarized in Figure 1.

### 3.1. Knowledge of Retrograde Peri-Implantitis Etiology

A total of 394 participants (82.9%) included correct answers about the etiology of retrograde peri-implantitis. Only the choice “marginal peri-implantitis becoming retrograde” was considered incorrect. A statistically significant difference in the number of correct answers was found out between the three groups for age (*p <* 0.001), years of experience in implant dentistry (*p =* 0.002) and number of implants placed per year (*p =* 0.002).

Implantologists belonging to the 31–44 years age group were more likely to (odds ratio: 13.16; 95% CI: 1.91–90.89) choose a correct response to the question on RPI etiology than those belonging to <30 group, while the group of implantologists placing < 20 implants/year were more likely to select the wrong answers (odds ratio: 0.316; 95% CI: 0.14–0.67) (Table 1).

### 3.2. Knowledge of Correct Answer on Radiographic Representation of Retrograde Peri-Implantitis

A total of 348 implantologists (73.3%) were capable of recognizing the correct radiographic representation of RPI (“a radiolucent area at the apical aspect of the implant”). A statistically significant difference in the number of correct answers was found between the three groups for years of experience in implant dentistry (*p* = 0.006) and number of implants placed/year (*p =* 0.005).

Implantologists belonging to the 10–19 years of experience group were more likely to (Odds ratio: 3.51; 95% CI: 1.14–10.77) choose a correct response to the question on RPI radiographic imaging than those belonging to the <5–9 years of experience group, while the group of implantologists placing < 20 implants/year (odds ratio: 0.416; 95% CI: 0.22–0.76) and between 20 and 80 implants/year (odds ratio: 0.530; 95% CI: 0.24–1.15) were more likely to select the wrong answers (Table 2).

### 3.3. Knowledge of Correct Answer on Symptoms of Retrograde Peri-Implantitis

A total of 388 implantologists (81.6%) recognized symptoms of RPI. The only incorrect choice was “implant mobility”. A statistically significant difference in the number of correct answers was found between the three groups just for number of implants placed per year (*p =* 0.035).

Implantologists belonging to the 31–44 years age group were more likely to (odds ratio: 2.19; 95% CI: 0.78–6.14) choose a correct response to the question on RPI symptoms than those belonging to the <30 group, while the group of implantologists placing 20–80 implants/year were more likely to select the wrong answers (odds ratio: 0.489; 95% CI: 0.22–1.04) (Table 3).

### 3.4. Knowledge of Correct Answer on Treatment of Retrograde Peri-Implantitis

A total of 306 implantologists (64.5%) excluded the incorrect choice “implant removal” from their answers. A statistically significant difference in the number of correct answers was found between the three groups based on the number of implants placed per year (*p =* 0.001).

Implantologists placing > 80 implants/year were more likely to (odds ratio: 2.55, 95% CI: 1.24–5.21) choose a correct response to the question on RPI treatment, while the group of implantologists belonging to the >45 years age group were more likely to select the wrong answers (odds ratio: 0.418, 95% CI: 0.15–1.11) (Table 4).

## 4. Discussion

The aim of the study was to evaluate the knowledge and attitude of Italian implantologists regarding retrograde peri-implantitis. To the best of the authors’ knowledge, this was the first study to report data on the perception and knowledge of clinicians on RPI.

Interestingly, even if all clinicians enrolled in this survey practiced implant dentistry, 26.7% of them ignored the radiographic representation of RPI (“a radiolucent area at the apical aspect of the implant”), selecting the wrong answers “a radio-opaque area at the apical aspect of the implant” and “a radiolucent line at the lateral side of the implant”.

Retrograde peri-implantitis could lead to implant failure if the periapical lesion extends to the marginal bone of the implant. Therefore, the ability of clinicians to recognize RPI as an apical radiolucent lesion surrounding the implant periapex is extremely important.

Regarding the origins of RPI, only 17.1% of participants included in their answers “marginal peri-implantitis becoming retrograde”, which is obviously incorrect. RPI is a disease entity affecting only the apical part of the implant and can destroy the circumferential bone [23]. As the exact etiology of RPI remains still controversial, all other answers (bone overheating during implant placement, presence of residual cystic cells, residual infection/inflammation of the tooth replaced by the implant or the endodontic lesion of neighboring teeth) were considered right.

RPI was defined by Quirynen et al. [20] as a clinically symptomatic periapical lesion, distinguished from the asymptomatic form, caused by over-drilling while preparing the implant osteotomy. In this clinical scenario, periapical implant lesions have the same radiographic aspect, but treatment is not necessary unless the size increases [22,32]. According to the results of the survey, 81.6% of clinicians are able to recognize RPI symptoms, without statistical differences between groups (*p* > 0.05). The answers included dull percussion, persistent pain, suppuration or fistula presence and no symptoms. The only answer considered wrong was “implant mobility”, because if present, an implant should always be considered as a failing implant to remove [33,34].

All treatment options of retrograde peri-implantitis should aim to prevent implant failure, leading to a re-osseointegration of the periapical implant area. There is no clear consensus on the ideal treatment option; however, authors [15,16] reported a 75–90% success rate in treatment of RPI, so the prognosis seems to be relatively favorable.

Therapeutic modalities can range from just prescribing an antibiotic therapy to the patient to surgical debridement of the apical part of the implant with/without the application of a bone substitute/guided bone regeneration (GBR) procedure and the possible resection of the apical part of the dental implant [35].

Of the respondents, 64.5% selected the following options: surgical debridement, surgical debridement + bone substitute/GBR and surgical debridement + bone substitute/GBR + apicoectomy of the implant, while implant removal was considered the wrong answer and was chosen by 35.5% of clinicians. Surgical debridement + bone substitute/GBR was the most selected option (24.4%).

Only 23.6% of the sample answered “yes” to the question “Have you have ever treated a RPI case?”; interestingly, 76.5% of them were > 45 years.

It is notable that for the question “Where did you learn about RPI?”, more than a quarter of the sample enrolled (26%) answered that they had never heard of RPI; 48.2% had > 45 years while 35.7% had more than 19 years of working experience; only 21.4% were <30 years. Only 9.8% answered “at the university”, and the majority of them were <30 years. The vast majority of participants (89.7%) who answered “for direct experience” were >45 years, with 72.4% of them with more than 19 years of working experience.

Based on the results of the study, incorrect answers were associated with less experienced participants (<80 implants/year) for all questions evaluated, with the exception of treatment strategies. Therefore, age and experience were associated with a higher number of correct answers and this could be explained by either their direct involvement in the diagnosis or treatment of RPI and their better knowledge of implant dentistry. Hence, more experienced and older implantologists have placed a greater number of dental implants throughout their career, compared to less experienced and younger clinicians, and faced, therefore, more complications. In contrast, as for the question on RPI treatment, survey takers > 45 years of age showed the worst results and this could probably be explained by their lower propensity for continuing education and scientific literature updates.

Major strengths of this survey are the great number of participants who completed the questionnaire and the use of multivariable regression to evaluate the statistical association between the selected variables. The response rate of 46.3% was considered adequate for a web questionnaire in order to represent the target population (Italian oral implantologists). The survey was anonymous to encourage answering the questions as truthfully as possible and to avoid the consequent risk of bias. Nevertheless, the authenticity of the answers collected was difficult to control, and another limitation was constituted by the same online nature of the survey, since it is uncertain whether participants’ answers matched their real behavior in clinical practice. Furthermore, since an interviewer was not present, there is no way to exclude the contemporary presence of multiple subjects at one computer or that respondents did not look for answers online.

## 5. Conclusions

Retrograde peri-implantitis is a pathological entity with an extremely low prevalence and has been scarcely investigated. Based on the results of our survey among implantologists, even if 26% of participants answered that they had never heard of RPI and only 23.6% of them have treated an RPI case, the vast majority of the sample was able to recognize symptoms and indicated the probable causes of RPI. On the other hand, surprisingly, 26.7% of the survey takers did not recognize radiographic representation of RPI, while 35.5% of participants picked “implant removal” when asked about treatment modality, showing a very limited knowledge of available management strategies and high survival rate after RPI surgical treatment. Within the limitations of this online survey, we can conclude that a better understanding of RPI is needed to promptly recognize periapical implant lesions and avoid implant removal, adopting an appropriate treatment.

Future research should be orientated in conducting international surveys to improve knowledge of retrograde peri-implantitis among clinicians.

## Figures and Tables

**Table 1:** Multivariable analysis considering the correct answers on the origin of retrograde peri-implantitis as the outcome variable.

		Correct Answers (%)	Odds Ratio	95% CI for Odds Ratio Lower Upper		*p*
Age (years)	≤30	84.2 ^a^				0.007
	31–44	98.2 ^b^	13.159	1.905	90.887	0.009
	≥45	77.7 ^a^	1.289	0.236	7.049	0.770
Years of experience in implant dentistry	<5–9	89.1 ^a^				0.593
	10–19	90.6 ^a^	0.664	0.126	3.490	0.629
	>19	77.3 ^b^	0.489	0.098	2.450	0.385
Number of implants placed in one year	>80	90.6 ^a^				0.011
	20–80	77.9 ^b^	0.495	0.176	1.394	0.183
	<20	89.3 ^a^	0.316	0.147	0.678	0.003

Each superscript letter (a,b) denotes a subset of categories whose column proportions do not differ significantly from each other at the 0.05 level.

**Table 2:** Multivariable analysis considering the correct answer on radiographic imaging for retrograde peri-implantitis.

		Correct Answers (%)	Odds Ratio	95% CI for Odds Ratio Lower Upper		*p*
Age (years)	≤30	81.6 ^a^				0.407
	31–44	78.2 ^a^	2.058	0.711	5.959	0.183
	≥45	70.2 ^a^	1.801	0.522	6.213	0.352
Years of experience in implant dentistry	<5–9	84.4 ^a^				0.031
	10–19	67.9 ^b^	3.515	1.147	10.771	0.028
	>19	71.1 ^b^	0.945	0.466	1.918	0.876
Number of implants placed in one year	>80	83.0 ^a^				0.018
	20–80	67.3 ^b^	0.530	0.242	1.158	0.111
	<20	78.6 ^a,b^	0.416	0.227	0.763	0.005

Each superscript letter (a,b) denotes a subset of categories whose column proportions do not differ significantly from each other at the 0.05 level.

**Table 3:** Multivariable analysis considering the correct answers on the symptoms of retrograde peri-implantitis.

		Correct Answers (%)	Odds Ratio	95% CI for Odds Ratio Lower Upper		*p*
Age (years)	≤30	81.6 ^a^				0.082
	31–44	87.3 ^a^	2.199	0.788	6.141	0.132
	≥45	81.0 ^a^	0.922	0.280	3.042	0.894
Years of experience in implant dentistry	<5–9	82.8 ^a^				0.307
	10–19	79.2 ^a^	0.929	0.350	2.464	0.882
	>19	84.5 ^a^	1.700	0.562	5.149	0.348
Number of implants placed in one year	<20	85.7 ^a^				0.102
	20–80	77.9 ^a^	0.489	0.229	1.044	0.065
	>80	88.7 ^a^	0.810	0.314	2.092	0.664

Each superscript letter (a,b) denotes a subset of categories whose column proportions do not differ significantly from each other at the 0.05 level.

**Table 4:** Multivariable analysis considering the correct answers on the treatment of retrograde peri-implantitis.

		Correct Answers (%)	Odds Ratio	95% CI for Odds Ratio Lower Upper		*p*
Age (years)	≤30	68.4 ^a^				0.217
	31–44	69.1 ^a^	0.635	0.297	1.357	0.241
	≥45	63.3 ^a^	0.418	0.157	1.111	0.080
Years of experience in implant dentistry	<5–9	63.1 ^a^				0.128
	10–19	71.2 ^a^	2.137	1.024	4.460	0.043
	>19	64.6 ^a^	1.718	0.716	4.123	0.226
Number of implants placed in one year	<20	64.3 ^a^				0.001
	20–80	58.7 ^a^	0.850	0.485	1.489	0.570
	>80	80.8 ^b^	2.551	1.247	5.216	0.010

Each superscript letter (a,b) denotes a subset of categories whose column proportions do not differ significantly from each other at the 0.05 level.

## Supplementary Materials

The following are available online at https://www.mdpi.com/1660-4601/17/22/8356/s1. Supplementary 1: Knowledge and attitude towards retrograde peri-implantitis among italian implantologists.

## References

[1] Anitua E., Piñas L., Alkhraisat M.H. (2016). Long-Term outcomes of immediate implant placement into infected sockets in association with immediate loading: A retrospective cohort study. J. Periodontol..

[2] Rossi F., Lang N.P., Ricci E., Ferraioli L., Baldi N., Botticelli D. (2018). Long-term follow-up of single crowns supported by short, moderately rough implants-A prospective 10-year cohort study. Clin. Oral Implants Res..

[3] Camps-Font O., Martín-Fatás P., Clé-Ovejero A., Figueiredo R., Gay-Escoda C., Valmaseda-Castellón E. (2018). Postoperative infections after dental implant placement: Variables associated with increased risk of failure. J. Periodontol..

[4] Papi P., Di Carlo S., Mencio F., Rosella D., De Angelis F., Pompa G. (2017). Dental Implants Placed in Patients with Mechanical Risk Factors: A Long-term Follow-up Retrospective Study. J. Int. Soc. Prev. Community Dent..

[5] Berglundh T., Armitage G., Araujo M.G., Avila-Ortiz G., Blanco J., Camargo P.M., Chen S., Cochran D., Derks J., Figuero E. (2018). Peri-implant diseases and conditions: Consensus report of workgroup 4 of the 2017 World Workshop on the Classification of Periodontal and Peri-Implant Diseases and Conditions. J. Periodontol..

[6] Papi P., Di Carlo S., Rosella D., De Angelis F., Capogreco M., Pompa G. (2017). Peri-implantitis and extracellular matrix antibodies: A case-control study. Eur. J. Dent..

[7] Renvert S., Persson G.R., Pirih F.Q., Camargo P.M. (2018). Peri-implant health, peri-implant mucositis, and peri-implantitis: Case definitions and diagnostic considerations. J. Periodontol..

[8] Papi P., Letizia C., Pilloni A., Petramala L., Saracino V., Rosella D., Pompa G. (2018). Peri-implant diseases and metabolic syndrome components: A systematic review. Eur. Rev. Med. Pharmacol. Sci..

[9] Di Murro B., Papi P., Passarelli P.C., D’Addona A., Pompa G. (2020). Attitude in radiographic post-operative assessment of dental implants among italian dentists: A cross-sectional survey. Antibiotics.

[10] Sussman H.I., Moss S.S. (1993). Localized osteomyelitis secondary to endodontic-implant pathosis. A case report. J. Periodontol..

[11] Marshall G., Canullo L., Logan R.M., Rossi-Fedele G. (2019). Histopathological and microbiological findings associated with retrograde peri-implantitis of extra-radicular endodontic origin: A systematic and critical review. Int. J. Oral Maxillofac. Surg..

[12] Schwarz F., Derks J., Monje A., Wang H.L. (2018). Peri-implantitis. J. Periodontol..

[13] Penarrocha-Diago M., Boronat-Lopez A., García-Mira B. (2009). Inflammatory implant periapical lesion: Etiology, diagnosis, and treatment presentation of 7 cases. J. Oral Maxillofac. Surg..

[14] Sarmast N.D., Wang H.H., Soldatos N.K., Angelov N., Dorn S., Yukna R., Iacono V.J. (2016). A Novel Treatment Decision Tree and Literature Review of Retrograde Peri-Implantitis. J. Periodontol..

[15] Peñarrocha-Oltra D., Blaya-Tárraga J.A., Menéndez-Nieto I., Peñarrocha-Diago M., Peñarrocha-Diago M. (2020). Factors associated with early apical peri-implantitis: A retrospective study covering a 20-year period. Int. J. Oral Implantol..

[16] Flanagan D. (2002). Apical (retrograde) peri-implantitis: A case report of an active lesion. J. Oral Implantol..

[17] McAllister B.S., Masters D., Meffert R.M. (1992). Treatment of implants demonstrating periapical radiolucencies. Pract. Periodontics Aesthet. Dent..

[18] Bain C.A., Moy P.K. (1993). The association between the failure of dental implants and cigarette smoking. Int. J. Oral Maxillofac. Implants.

[19] Zhou W., Han C., Li D., Li Y., Song Y., Zhao Y. (2009). Endodontic treatment of teeth induces retrograde peri-implantitis. Clin. Oral Implants Res..

[20] Quirynen M., Vogels R., Alsaadi G., Naert I., Jacobs R., van Steenberghe D. (2005). Predisposing conditions for retrograde peri-implantitis, and treatment suggestions. Clin. Oral Implants Res..

[21] Reiser G.M., Nevins M. (1995). The implant periapical lesion: Etiology, prevention, and treatment. Compend. Contin. Educ. Dent..

[22] Temmerman A., Lefever D., Teughels W., Balshi T.J., Balshi S.F., Quirynen M. (2014). Etiology and treatment of periapical lesions around dental implants. Periodontol. 2000.

[23] Park S.H., Sorensen W.P., Wang H.L. (2004). Management and prevention of retrograde peri-implant infection from retained root tips: Two case reports. Int. J. Periodontics Restor. Dent..

[24] Saleh M.H.A., Khurshid H., Travan S., Sinjab K.H., Bushahri A., Wang H.L. (2020). Incidence of retrograde peri-implantitis in sites with previous apical surgeries: A retrospective study [published online ahead of print, 2020 May 25]. J. Periodontol..

[25] Rosella D., Papi P., Pompa G., Capogreco M., De Angelis F., Di Carlo S. (2017). Dental students’ knowledge of medication-related osteonecrosis of the jaw. Eur. J. Dent..

[26] Ong A., Kim J., Loo S., Quaranta A., Rincon A.J.C. (2019). Prescribing trends of systemic antibiotics by periodontists in Australia. J. Periodontol..

[27] Rodríguez Sánchez F., Arteagoitia I., Rodríguez Andrés C., Caiazzo A. (2019). Antibiotic prophylaxis habits in oral implant surgery among dentists in Italy: A cross-sectional survey. BMC Oral Health.

[28] Quaranta A., Andreana S., Pompa G., Procaccini M. (2014). Active implant peri-apical lesion: A case report treated via guided bone regeneration with a 5-year clinical and radiographic follow-up. J. Oral Implantol..

[29] Piattelli A., Scarano A., Balleri P., Favero G.A. (1998). Clinical and histologic evaluation of an active "implant periapical lesion": A case report. Int. J. Oral Maxillofac. Implants.

[30] Scarano A., Di Domizio P., Petrone G., Iezzi G., Piattelli A. (2000). Implant periapical lesion: A clinical and histologic case report. J. Oral Implantol..

[31] Piattelli A., Scarano A., Piattelli M., Podda G. (1998). Implant periapical lesions: Clinical, histologic, and histochemical aspects. A case report. Int. J. Periodontics Restor. Dent..

[32] Chieruzzi M., Pagano S., De Carolis C., Eramo S., Kenny J.M. (2015). Scanning Electron Microscopy Evaluation of Dental Root Resorption Associated with Granuloma. Microsc. Microanal..

[33] Piattelli A., Scarano A., Favero L., Iezzi G., Petrone G., Favero G.A. (2003). Clinical and histologic aspects of dental implants removed due to mobility. J. Periodontol..

[34] Zetterqvist L., Feldman S., Rotter B., Vincenzi G., Wennström J.L., Chierico A., Stach R.M., Kenealy J.N. (2010). A prospective, multicenter, randomized-controlled 5-year study of hybrid and fully etched implants for the incidence of peri-implantitis. J. Periodontol..

[35] Ramanauskaite A., Juodzbalys G., Tözüm T.F. (2016). Apical/Retrograde Periimplantitis/Implant Periapical Lesion: Etiology, Risk Factors, and Treatment Options: A Systematic Review. Implant Dent..

