# Supplementary material for: Knowledge and Attitude towards Retrograde Peri-Implantitis among Italian Implantologists: A Cross-Sectional Survey
Source: Int J Environ Res Public Health. 2020 Nov 12;17(22):8356. doi: 10.3390/ijerph17228356 (PMC7696335; doi:10.3390/ijerph17228356)
Supplement: Supplementary file 1 [file ijerph-17-08356-s001.pdf]

KNOWLEDGE AND ATTITUDE TOWARDS RETROGRADE PERI-IMPLANTITIS AMONG ITALIAN IMPLANTOLOGISTS

Age: $\leq$30 31-44 $\geq$45

Sex: M F

Years of experience in dentistry: < 5 5-9 10-19 >19

Do you perform implant dentistry on a regular basis? YES NO

Number of dental implants placed per year <20 20- 80 >80

1 Where did you learn about retrograde peri-implantitis?
   a) at the University
   b) From scientific papers
   c) From direct experience
   d) For colleagues' experience
   e) I've never heard of it

2) What are the causes of RPI? (more than one answer is allowed)
   a) Bone overheating during implant drilling
   b) presence of residual cystic cells
   c) residual infection/inflammation of the tooth replaced by the implant
   d) marginal peri-implantitis becoming retrograde
   e) periapical endodontic lesions of neighbouring teeth

3) Which is the radiographic representation of RPI?
   a) a radio-opaque area at the apical aspect of the implant
   b) a radiolucent area at the apical aspect of the implant
   c) a radiolucent line at the lateral side of the implant

4) What are the symptoms of RPI? (more than one answer is allowed)
   a) Dull percussion
   b) Persistent pain
   c) Implant mobility
   d) Suppuration and/or presence of fistula
   e) None

5) How would you treat an implant affected by RPI? (more than one answer is allowed)
   a) surgical debridement
   b) surgical debridement + bone substitute/GBR
   c) surgical debridement + bone substitute/GBR + apicoectomy of the implant
   d) implant removal

6) Have you have ever treated a RPI case?
   a) yes
   b) no
